# A novel Slide-seq based image processing software to identify gene expression at the single cell level

**DOI:** 10.1016/j.jpi.2024.100384

**Published:** 2024-05-31

**Authors:** Th.I. Götz, X. Cong, S. Rauber, M. Angeli, E.W. Lang, A. Ramming, C. Schmidkonz

**Affiliations:** a Department of Nuclear Medicine, University Hospital Erlangen, 91054 Erlangen, Germany; b CIML Group, Biophysics, University of Regensburg, 93040 Regensburg, Germany; c Department of Internal Medicine, University Hospital Erlangen, Erlangen, Germany; d Department of Industrial Engineering and Health, Technical University of Applied Sciences Amberg-Weiden, Weiden, Germany

**Keywords:** Cell segmentation, Pruning, Deep neural networks, Slide-seq, DNN complexity

## Abstract

Analysis of gene expression at the single-cell level could help predict the effectiveness of therapies in the field of chronic inflammatory diseases such as arthritis. Here, we demonstrate an adopted approach for processing images from the Slide-seq method. Using a puck, which consists of about 50,000 DNA barcode beads, an RNA sequence of a cell is to be read. The pucks are repeatedly brought into contact with liquids and then recorded with a conventional epifluorescence microscope. The image analysis initially consists of stitching the partial images of a sequence recording, registering images from different sequences, and finally reading out the bases. The new method enables the use of an inexpensive epifluorescence microscope instead of a confocal microscope.

## Introduction

Mapping gene expression at the single-cell level within tissues remains a technical challenge.^2^^,^^18^ Measurement of the location of molecules in tissues is essential for understanding tissue formation and function defining molecular pathways involved in several disease states.^6^^,^^10^ The identification of spatially defined gene expression patterns can provide insights into the development and maintenance of complex tissue architectures and the molecular characterization of pathological states. Formerly, technologies for spatially encoded RNA sequencing with barcoded oligonucleotide capture arrays were limited to resolutions in hundreds of micrometers limiting the detection of tissue features.^15^ Rodrigues et al. developed the Slide-seq method which enables the transcriptome-wide detection of RNAs with a spatial resolution of 10 μm.^14^ In Slide-seq, freshly frozen tissue can be sliced onto prepared arrays of DNA-barcoded beads termed pucks, causing RNA in the tissue to transfer onto the beads. Subsequent library preparation yields data that are equivalent to single cell RNA sequencing data, but with a spatial location associated with each bead. The authors herewith presented a scalable method for obtaining spatially resolved gene expression data at resolutions that are comparable to the size of individual cells. In contrast to imaging-based transcriptomics that enable the identification of preselected genes in fixed specimens, array-based approaches decouple the imaging from molecular sampling and allow for transcriptome-wide identification of molecular patterns in tissue sections.^19^ Because Slide-seq's low transcript detection sensitivity limited the applicability to different disease states, Stickels et al. presented a novel protocol, termed Slide-seqV2.^16^ This improved method demonstrated an order of magnitude higher sensitivity by improving bead synthesis and array indexing to reach an RNA capture efficiency of about 50% of that of single-cell RNA-seq data. Slide-seq can be easily integrated with large-scale scRNA-seq datasets and can facilitate the discovery of spatially defined gene expression patterns in normal and diseased tissues at reduced costs.^14^ Commercially available spatial trancriptomic arrays are expensive, the readout demands on high computational power and data processing is very slow.^17^^,^^20^ Therefore, the use of such arrays is very limited. On the other hand, the functional heterogeneity of inflammatory cells exhibit a high degree of spatial organization that influences the local microenvironment, i.e., whether it supports inflammation or allows switching into resolution of inflammation.^1^^,^^3^^,^^11^ In arthritis, the lining and sublining compartments of the synovium are particularly prominent, with the sublining compartment having a high degree of diversity containing pro-inflammatory and pro-resolving cell types.^4^^,^^5^^,^^9^ The spatiotemporal distribution of cells and gene expression is therefore of interest for understanding the spatial orchestration of resolution processes in arthritis. To date, scRNAseq following tissue dissociation has provided unbiased insights into the cellular composition of synovial tissue, but it lacks information about cellular neighbourhood and segregations.^21^^,^^22^ The goal of our work was to establish a complete image processing pipeline to identify gene expression at the single cell level by using machine learning algorithms and conventional epifluorescence microscopy to reduce costs and increase the availability of this approach.

## Method

In this study, we present a methodology to process images collected by the Slide-seq method. The latter transfers RNA from tissue sections onto a surface covered with DNA barcode beads with known positions, allowing the locations of the RNA to be deduced by sequencing. In cell analysis, so-called pucks, with a size of 3 mm, are applied to a carrier material. A puck consists of about 50,000 DNA barcode beads, which carry attached RNA sequences. These pucks are successively brought into contact with 14 different liquids and then different fluorescence images and one brightfield image are taken. After the puck has been sequenced, a cell sample is applied and the RNA sequence is read out. Further details of the exact procedure can be found here.^14^

### Data set

**Fig. 1:**
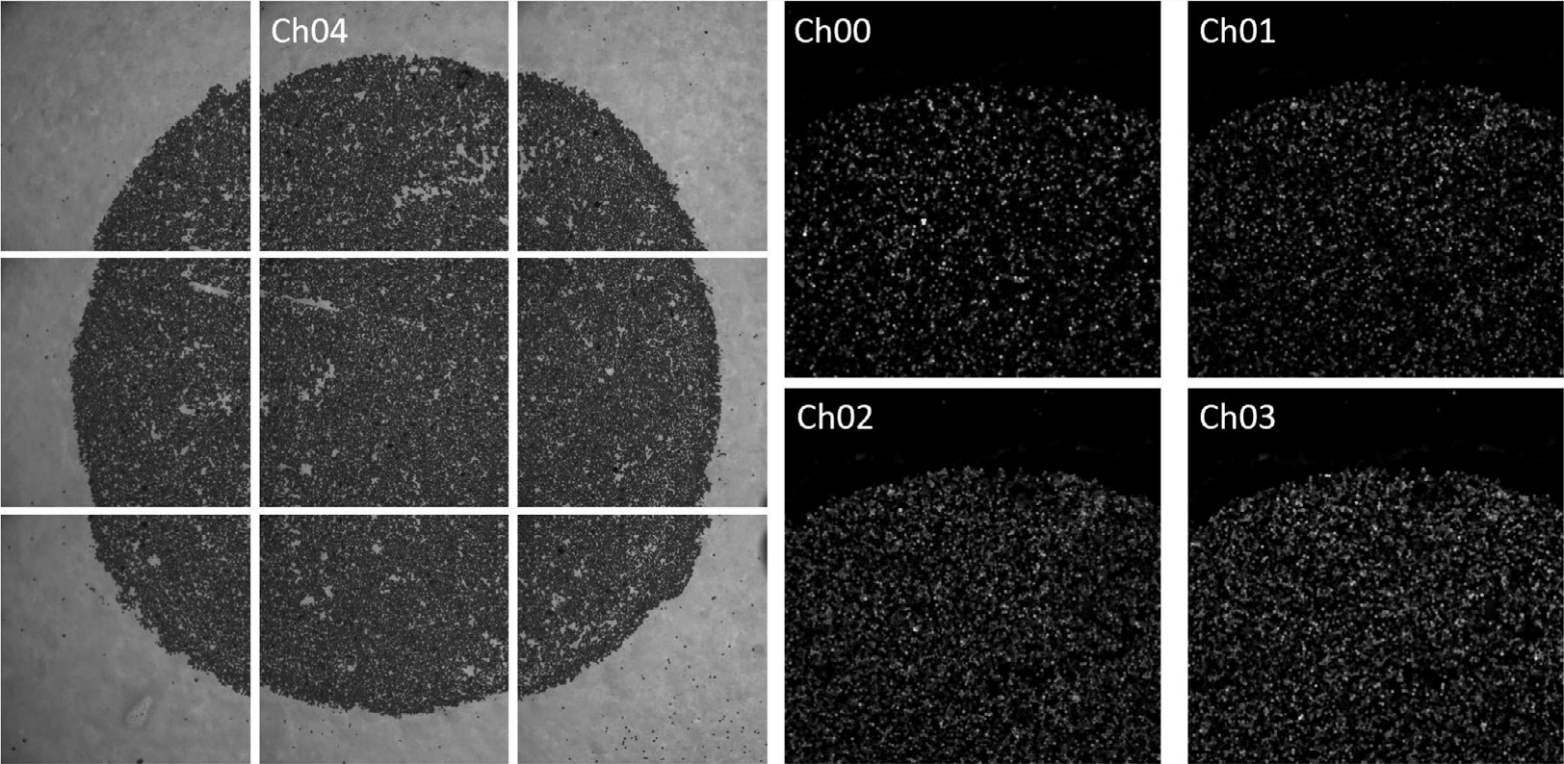
Left: Nine partial images of one puck in the visible light range (image size 1024×1024), Right: four fluoreszenz channels of one partial image.

For this study, 14 images were taken from each of seven different pucks with a THUNDER Imager Live Cell and 3D Assay from Leica Microsystems. Each of these 14 images consists of nine partial images due to the limited field of view of the microscope (see Fig. 1). The spatial resolution is 1024×1024 pixels per partial image. Nine partial images depict the entire puck. Each partial image is recorded with four different fluorescence wavelengths (475 nm, 555 nm, 575 nm, 635 nm) and an additional recording is taken in the visible light range. By adding hybridization buffers containing different dye-conjugated oligonucleotides, beads carrying different DNA bases interact with different oligonucleotides, hence emit fluorescent light at different wavelengths. Consequently, depending on the fluorescence channel in which a bead is visible, the base it is carrying can be identified easily. Additionally, an image of a puck was acquired in an epifluorescence microscope and a confocal microscope to generate an image data set that was well suited to train neural network algorithms. These image data sets are referred with AddImg in the following. An overview of the acquired images is given in Table 1.

**Table 1:** In this table, an overview of the acquired images is given.

Images per puck					
Channels	Bright field	475 nm	555 nm	575 nm	635 nm
Partial images one sequence	9	9	9	9	9
Partial images 14 sequences	126	126	126	126	126
Images of seven puck					
Channels	Bright field	475 nm	555 nm	575 nm	635 nm
Partial images	63	63	63	63	63
Partial images 14 sequences	882	882	882	882	882
Images of two microscopes (AddImg)					
Channels	Bright field	475 nm	555 nm	575 nm	635 nm
Partial images Thunder	9	9	9	9	9
Channels	Bright field	405 nm	488 nm	552 nm	638 nm
Partial images Confocal	9	9	9	9	9
Partial images 14 sequences	Only one sequence was acquired				

**Fig. 2:**
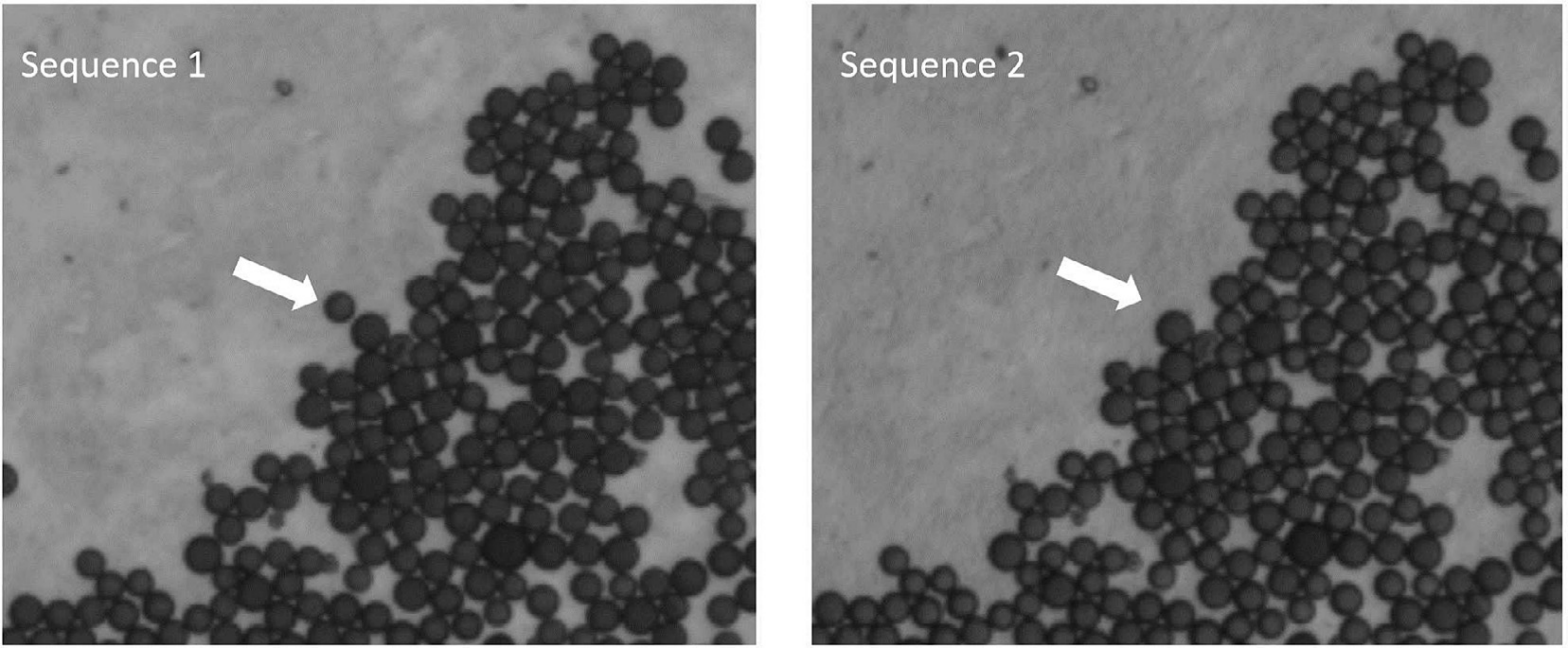
Same puck region in two different sequence images (image size 50×50). The bead, which was washed away is marked with an arrow.

The aim of this project is to identify a characteristic sequence of 14 bases for each of the roughly 50,000 beads. This, however, presupposes that the same bead can be identified in different images. But probe handling introduces a severe obstacle here: The pucks need to be brought into contact with liquids containing different dyes. Fourteen sequences need to be performed for each puck. Between the sequences, the puck is removed from the microscope and washed with a dye-containing liquid before it is placed back in the microscope. Each time, this changes the position of the puck relative to the microscope and renders the taken images incongruent. In addition, some beads are washed away by the application of liquid (see Fig. 2). Finally, the gray values of the beads are varying between the sequences and the partial images because of various characteristics of the optical mapping. All these shortcomings render data processing rather challenging.

### Image statistics

Image statistics were evaluated with the goal to find out how evenly the beads are distributed over the puck, how often beads are washed off and in which sequence. First manual evaluations were carried out to quantify the mismatch between images of a sequence. The same area of every puck was cut out for each sequence, allowing differences in the number of beads and their positional changes to be identified across the 14 sequences. For the first puck, 1288 partial images for all 14 sequences were divided into small image patches containing 50×50 pixels. The size of this image section is sufficient to identify changes between the two sequences. However, using larger sections resulted in poorer change detection because of reduced clarity. Only the visible beads that were completely within the partial image section were counted for each image patch.

In addition, manual evaluations were used to train the learning system. For the localization of the beads, a segmentation of all beads in one partial image was required. To generate the segmentation, a partial image of the epifluorescence microscope was taken from the AddImg data set. During segmentation, the center points of the beads were identified while maintaining a 2–3 pixel margin from the boundary of the beads.

Manual labeling on the AddImg data set was also required for the estimation of the bases in the epifluorescence microscope. Estimation was based on the use of the gray values of the fluorescence images in the epifluorescence microscope. For this purpose, the associated beads were searched for in the epifluorescence microscope image for 1746 beads in an image section in the confocal microscope and the respective gray values in the fluorescence image were saved together with the color value in the confocal image. Because spectral separation in the confocal microscope is much higher, the thereby estimated base is taken as ground truth.

Manual analyses were also performed to estimate algorithm accuracy. With the algorithm presented in this work, a base sequence per bead can be determined. For this, it is essential that the same bead is identified in all 14 sequence images. In order to check this, 1000 determined bead positions in 14 sequences each were checked visually for correctness.

### Image analysis

The image analysis can be divided into the following steps:

• Preprocessing of partial images
– Calculation of binary mask
– Image stitching
– Rough registration
• Acurate registration
• Base estimation

#### Preprocessing of partial images

**Fig. 3:**
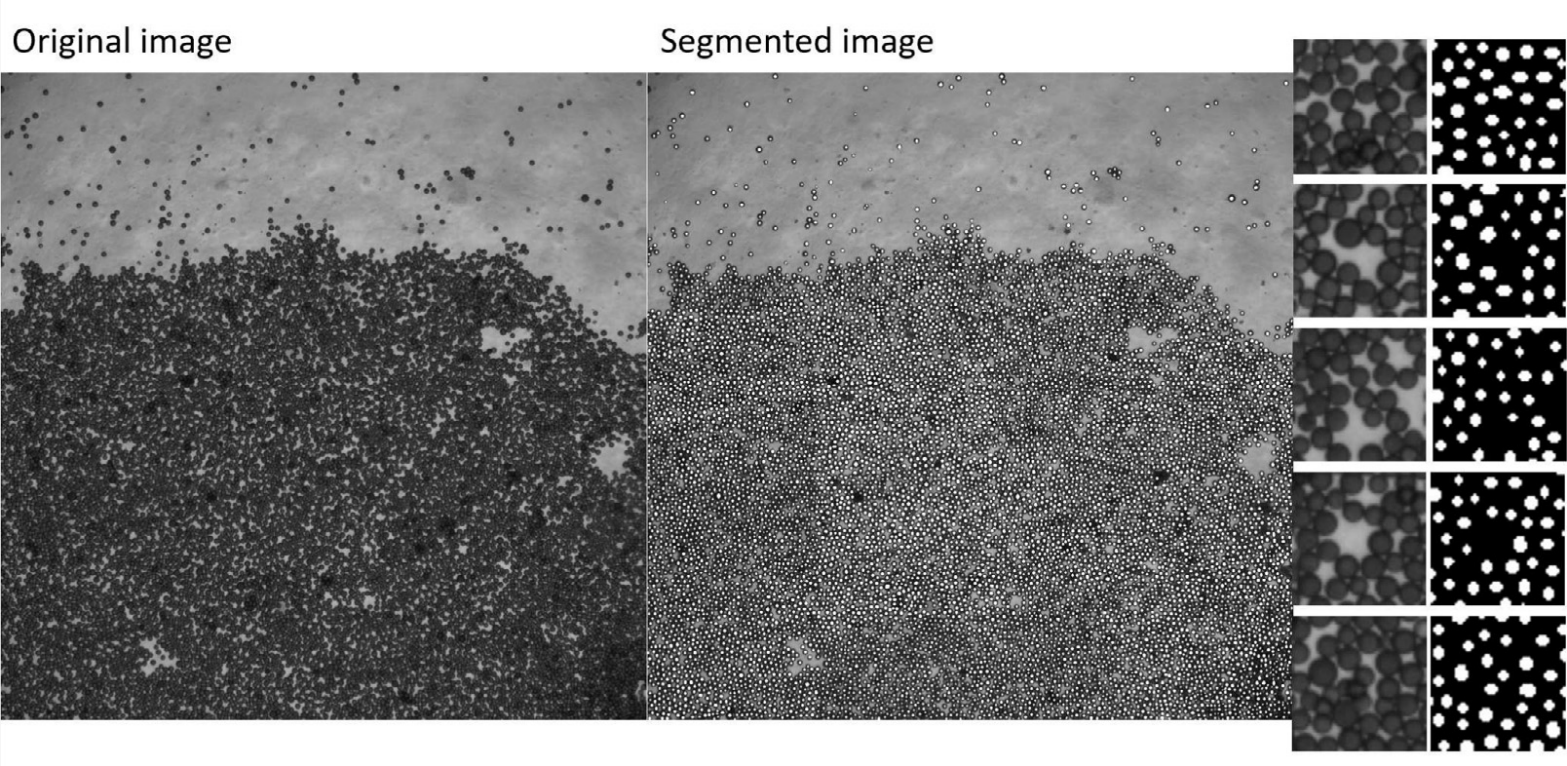
On the left side, the original image and the manually segmented is shown and on the right side, the croped patches used for training.

**Fig. 4:**
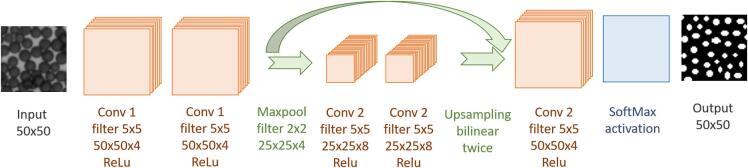
The architecture of the neural network for the rough segmentation of the single beads.

Due to the different puck positions when taking an image repeatedly, identical beads have different gray values in the individual images. Hence, optimizing gray value differences between pairs of beads across a sequence of images is meaningless. For this reason, a binary mask was first calculated from the images in the visible light range. To do so, for one image, we segmented manually all 9824 visible beads. Afterwards, a neural network was trained with cropped image patches of dimension 50×50 pixels (see Fig. 3). The network architecture is shown in Fig. 4. For training, an ADAM optimizer was used and the binary cross-entropy was chosen as loss function. Only the central area of the bead has been segmented by the small neural network, and not the borders, because the segments should not overlap. From the single segments, the center point for each bead can be estimated by taking the mean position of each segment. At each position, a circle with a defined radius is generated in an empty image.

The binary masks make it easier to stitch and register the images. The next step is the stitching of the sub-images into a large overall image. From the image recording with the microscope, it is known that the overlap of two images is 10% of the image size. First, two horizontally adjacent images are selected and the sum of the positional differences in the overlapping region is calculated. This serves as an optimization function. The position of the second image is shifted horizontally until the minimum value is reached. Six of these pairs of images exist in the horizontal direction. Once the relative displacement of two images is known, an overall image can be created. This is not yet optimized, especially in the vertical direction. However, in order to achieve an optimal alignment in both horizontal and vertical directions, all sub-images, except for the first one, are again shifted in the x- and y direction, thus optimizing the sum of the differences of all overlapping areas. Hence, the algorithm performs a grid search. After the stitch of the partial images, a very good image of the entire puck is obtained. Still image errors can be detected at the boundary regions, which result from optical distortions and a varying magnification, leading to a tilt of the optical axis.

The beads on the pucks are not evenly distributed like in a grid structure. This effect helps with image registration. The latter is necessary to identify the positions of individual beads in all images. First of all, the center of all known bead positions is calculated for the stitched mask and this center of the mask is shifted to the origion of the coordinate system. After centering the image, the centers of the pucks lie very well on top of each other, so it is sufficient to primarily optimize the rotation angle. After a suitable rotation has been determined, the displacement in the x- and y directions and the exact angle of rotation can be determined again by a grid search. To speed it up, the resolution of the images has been reduced by a factor of two for the optimization process. The sum of the differences between the two stitched masks is used as an optimization function. The puck position of the first image serves as a reference for all further puck images.

#### Accurate registration

After the first registration, the puck images lie very well on top of each other, but the positions of the individual beads deviate from each other due to imaging errors during the image recording. For this reason, a fine adjustment is necessary. The two puck images to be registered are broken down into small image patches. The bead positions contained in each patch are known. The patch will be shifted till the sum of distances between the points is minimized. If the difference between two points in the different patches is less than four pixels, the points will be considered as being congruent. With this method, points of all other sequences can be assigned for each point of the first sequence.

#### DNA base estimation

**Fig. 5:**
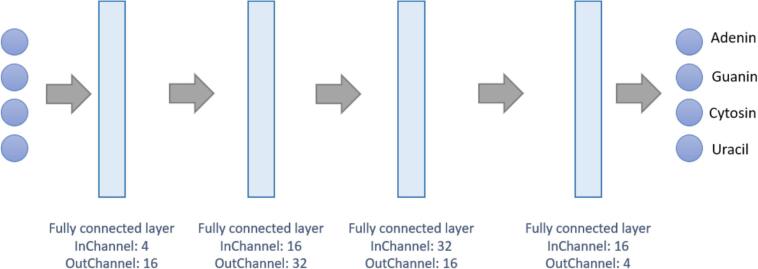
The architecture of the neural network for the estimation of the base based on the gray values of the fluorescence images.

After the fine adjustment, the position of each individual bead in the 14 puck images is known, so the related DNA base can be assigned from the fluorescence images. In comparison to the confocal microscope, the spectral channels (RGB) of the epifluorescence microscope are not clearly separated from each other, and so there is cross-talk between the image channels. Since a threshold value definition is not possible due to the different dye intensities, a neural network was trained to predict the DNA bases from the fluorescence images. The network architecture is shown in Fig. 5. The ADAM optimizer was used with a learning rate of 0.001 and a mean squared error loss function was deployed. Altogether1575 data samples were used for training and 171 for testing. The training data set for the network training was generated as follows: The dominant color of 1746 beads was read manually from the images of the confocal microscope and the corresponding beads were identified in the epifluorescence microscope image. Based on the dominating image intensity in the four fluorescence channels of the confocal microscope, the network learns to identify one of the four assigned DNA bases.

## Results and discussion

### Image statistics

**Fig. 6:**
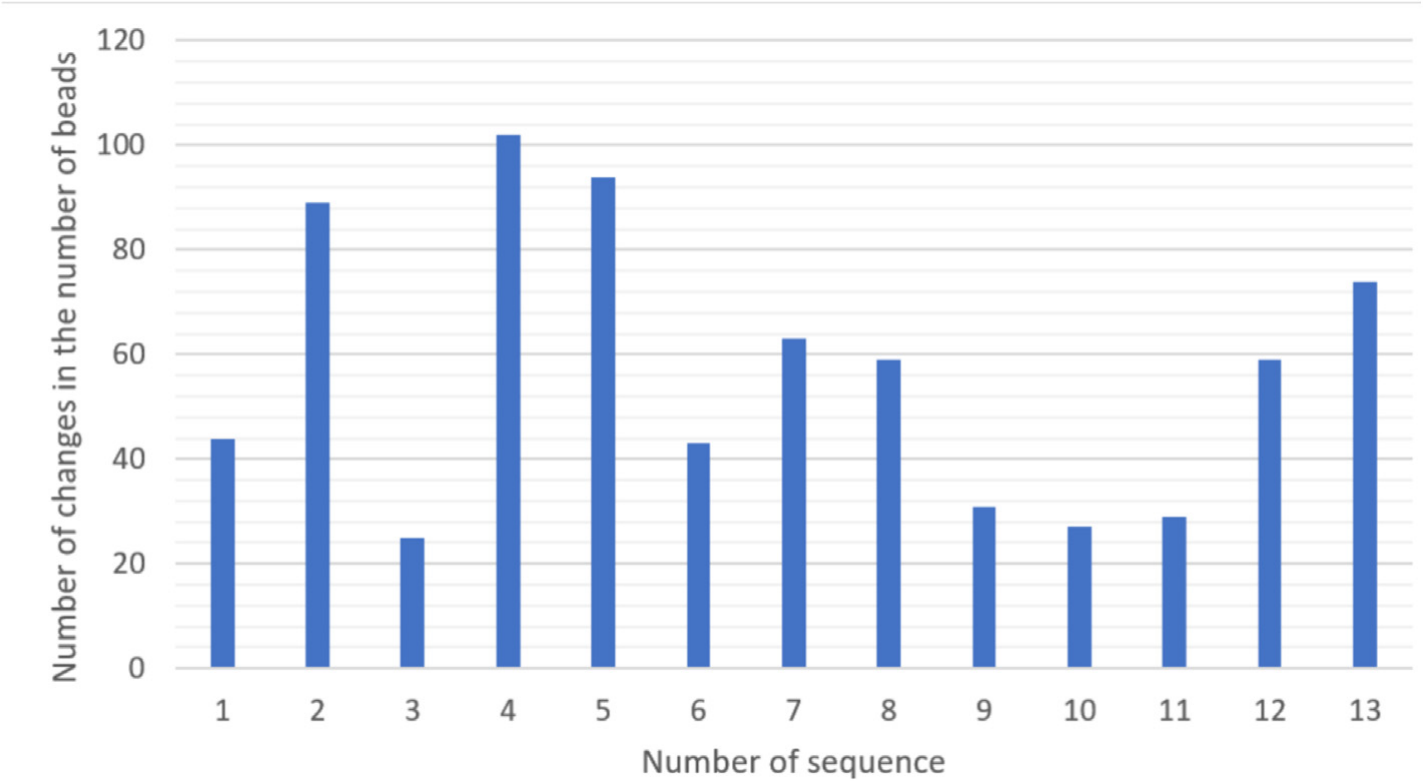
Frequency of the changes in the number of beads per sequence.

Visual inspection of all sequences from one puck revealed that 428 patches out of 1288 had changes in the number of beads in the frame. For Fig. 6, the number of changes in the total number of beads per patch for each sequence is shown for all 1288 images. The most frequent changes occurred in sequences 2,4,5,13. These sequences were imaged last. Therefore, the beads become a bit unstable on the puck. The beads are attached to the carrier material using an adhesive. After multiple washing processes, individual beads can leave their place. The most common change in the sequences is the disappearance or addition of a bead.

### Preprocessing of partial images

**Fig. 7:**
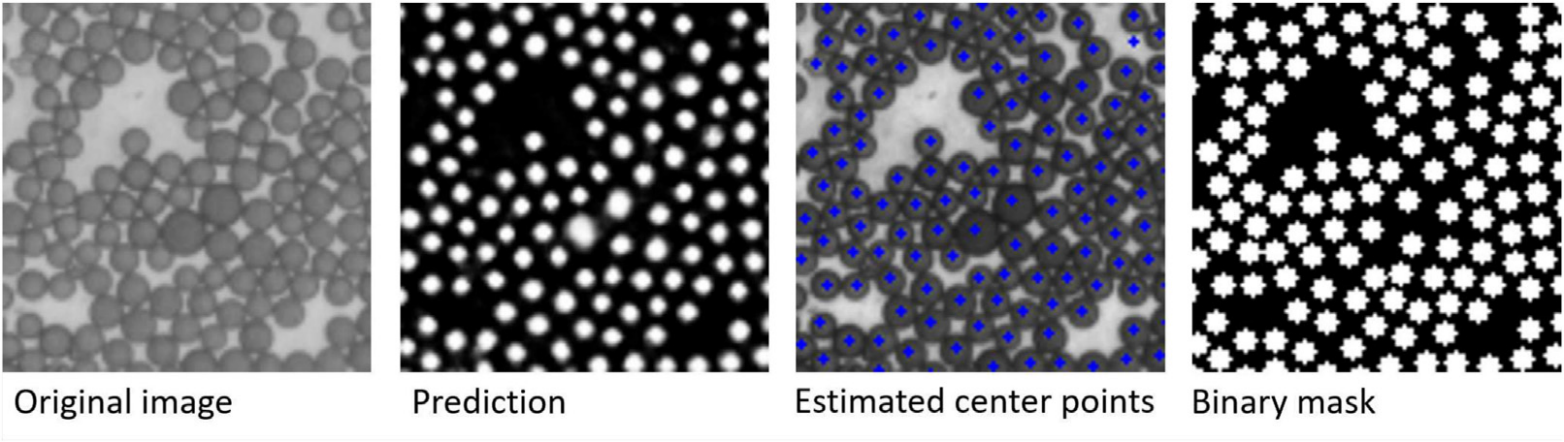
Single steps of the process of binary mask generation.

First, a binary mask has to be created in order to become independent of any optical distortions of incident light during image recording. A neural network receives the image in the visible light range as input and uses it to estimate a rough segmentation of the beads. Based on the segmented beads, the position of their center point can be calculated for each segment. Then, in an empty image, a corresponding circle with a radius of three pixels is drawn at all identified bead positions. The individual steps of mask generation are highlighted in Fig. 7. These steps are repeated for each of the nine partial images.

**Fig. 8:**
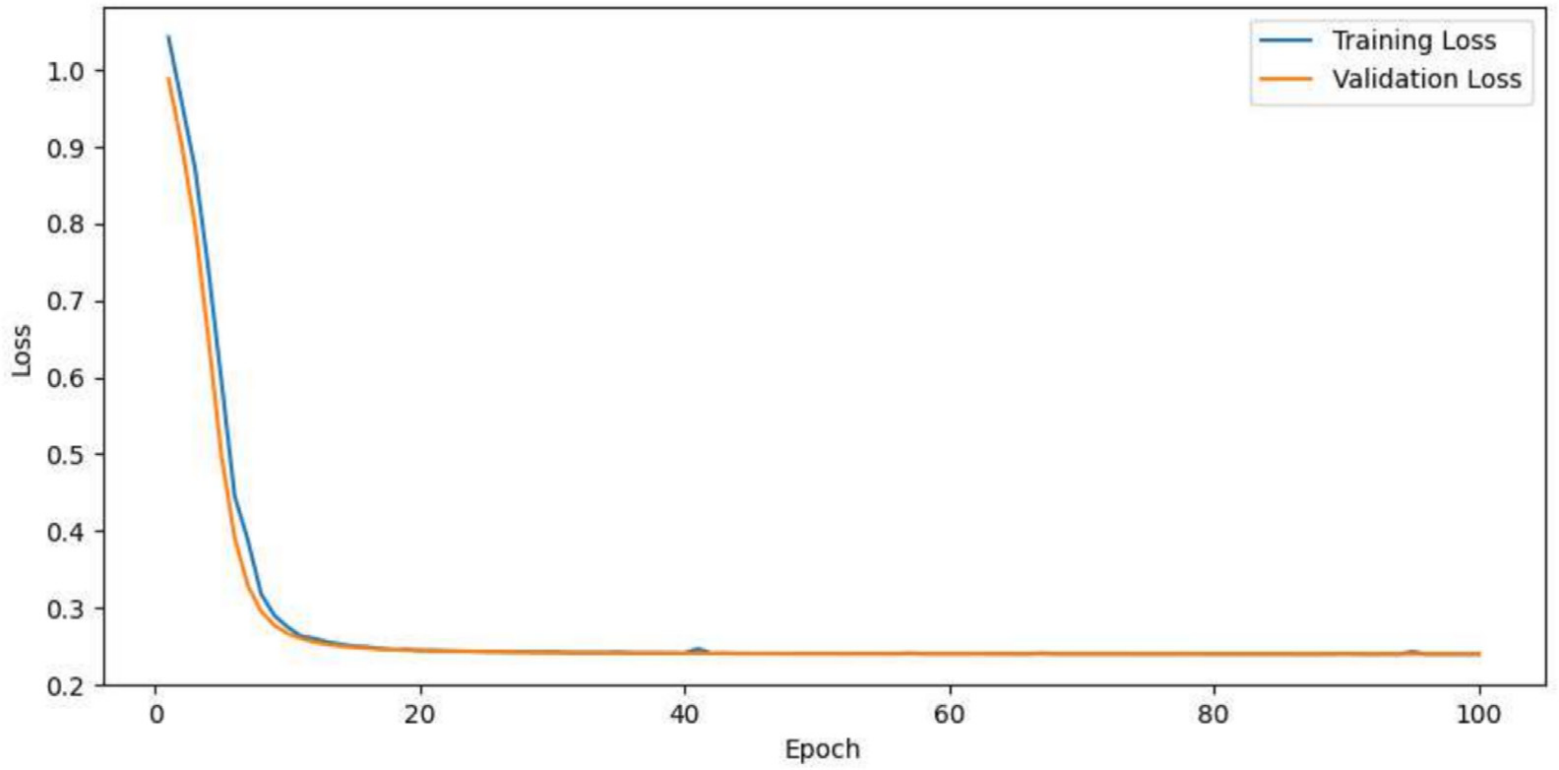
Training and validation loss for the neural network over the epochs.

Although the neural network was only trained using an image data set to create the segmentation, it is able to generate a very precise segmentation. The reason for this is that the network architecture is chosen to be as small as possible, so that even a small number of training examples is sufficient for the network to generalize.^7^^,^^13^ In Fig. 8, the training and validation loss for the training process is shown. A binary image is created by calculating the center point and then generating uniformly sized points.

**Fig. 9:**
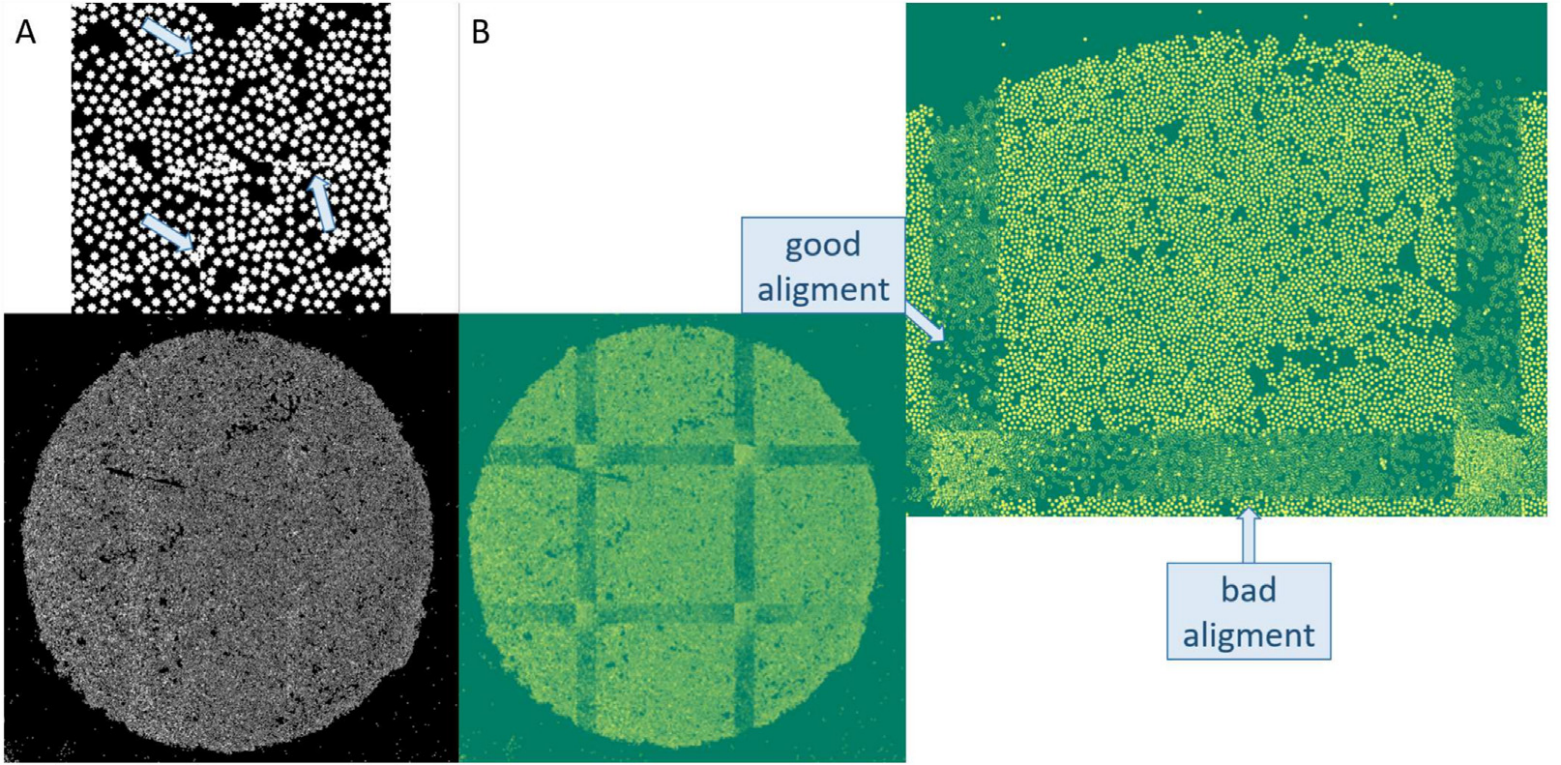
(A) Stitched binary mask with a zoomed region, where the stitching errors are marked with blue arrows. (B) The stitched images, where the absolute difference between the partial images was calculated, therefore the overlapping regions can be sawn clearly. In a perfect scenario, the overlap regions would be dark green. (For interpretation of the references to color in this figure legend, the reader is referred to the web version of this article.)

Next, the nine sub-images were stitched together to form an entire image. The related stitching algorithm minimizes the sum of the positional differences in the overlap region. The stitched mask is shown in Fig. 9. In the zoomed-in area, small errors can be seen at the boundaries. In order to show the overlapping regions better, the pixel intensities of the sub-images were subtracted from each other when composing the image, and the absolute amount was taken. As a result, a perfect overlap would be represented as a homogeneous green stripe resulting from the overlap of the underlying RGB channel colors. Looking more closely at the overlapping regions, it becomes clear that some border areas match very well, whereas others exhibit larger deviations. In addition, individual yellow beads can be seen, which are then only present in one of the two images. The stitching process is performed for all 14 sequences.

Whereas lens distortion poses a significant hurdle for precision measurements in microscopy, current correction techniques are limited by their need for detailed knowledge about the microscope's lens design. This requirement makes them impractical in many situations.^8^^,^^12^ The algorithm presented is developed in such a way that it is able to process the images despite optical distortions.

**Fig. 10:**
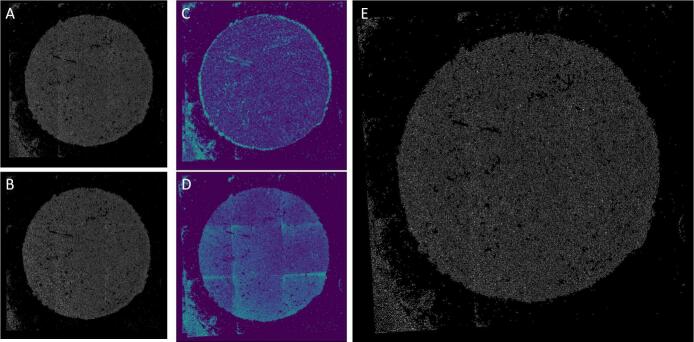
(A) Stitched mask from the first sequence. (B) Stitched mask from the second sequence. (C) Absolute difference image of images A and B before registration. (D) Absolute difference image of images A and B after registration. (E) Tranformed image B after registration.

After an entire image of all sequences has been registered, the former can be registered on top of each other. The first recorded sequence serves as a reference for the others. The global images of each sequence are centered, which means that the centers of the pucks are already very well aligned. Next, the angle of rotation is optimized. The displacement in the x- and y direction is then varied in ever smaller increments and smaller angles of rotation are also tried out. The result is a very good registration of the two puck images (see Fig. 10).

After the global images have been co-registered, small image patches were then optimized. In order to check whether the same bead was detected in each sequence, 1000 bead positions in 14 sequences each were checked manually. Overall, 90% of the beads were correctly assigned in all 14 sequences.

The last step of the method is to estimate the DNA base assigned to each bead. Therefore, a neural network was trained to estimate the DNA base with respect to the gray values of the four fluorescence images. The network was trained for 100 epochs and tested on the test set. The overall accuracy of the prediction was 98.2%. Consequently, the optical assignment of a DNA base is possible only using an epifluorescence microscope, a confocal microscope is not needed. This result helps researchers to save quite some time and money.

## Conclusion

We presented an adopted approach for analysing gene expressions at the single-cell level from the Slide-seq method. The image processing pipeline extracts RNA sequences of cells from roughly 50,000 DNA barcode beads. The new method enables the use of an inexpensive epifluorescence microscope instead of a confocal microscope. Hence, it offers an efficient and less complex toll for spatially resolved transcriptomics than existing alternatives.

## Declaration of competing interest

There is no conflict of interest.

## References

[1] Brockmann L., Soukou S., Steglich B. (2018). Molecular and functional heterogeneity of il-10-producing cd4+ t cells. Nat Commun.

[2] Chen K.H., Boettiger A.N., Moffitt J.R., Wang S., Zhuang X. (2015). Spatially resolved, highly multiplexed rna profiling in single cells. Science.

[3] Cormican S., Griffin M.D. (2020). Human monocyte subset distinctions and function: insights from gene expression analysis. Front Immunol.

[4] Donlin L.T., Park S.-H., Giannopoulou E. (2019). Insights into rheumatic diseases from next-generation sequencing. Nat Rev Rheumatol.

[5] Ermann J., Rao D.A., Teslovich N.C., Brenner M.B., Raychaudhuri S. (2015). Immune cell profiling to guide therapeutic decisions in rheumatic diseases. Nat Rev Rheumatol.

[6] Jung H., Gkogkas C.G., Sonenberg N., Holt C.E. (2014). Remote control of gene function by local translation. Cell.

[7] Lanouette R., Thibault J., Valade J.L. (1999). Process modeling with neural networks using small experimental datasets. Comput Chem Eng.

[8] Liu X., Li Z., Zhong K., Chao Y.J., Miraldo P., Shi Y. (2018). Generic distortion model for metrology under optical microscopes. Opt Lasers Eng.

[9] Mulherin D., Fitzgerald O., Bresnihan B. (1996). Synovial tissue macrophage populations and articular damage in rheumatoid arthritis. Arthr Rheumat Off J Am Coll Rheumatol.

[10] Munsky B., Neuert G., Van Oudenaarden A. (2012). Using gene expression noise to understand gene regulation. Science.

[11] Nathan C., Ding A. (2010). Nonresolving inflammation. Cell.

[12] Olivo J.C., Kahn E., Halpern S., Fragu P. (1991). Image registration and distortion correction in ion microscopy. J Microsc.

[13] Pasini A. (2015). Artificial neural networks for small dataset analysis. J Thoracic Dis.

[14] Rodriques S.G., Stickels R.R., Goeva A. (2019). Slide-seq: a scalable technology for measuring genome-wide expression at high spatial resolution. Science.

[15] Ståhl P.L., Salmén F., Vickovic S. (2016). Visualization and analysis of gene expression in tissue sections by spatial transcriptomics. Science.

[16] Stickels R.R., Murray E., Kumar P. (2021). Highly sensitive spatial transcriptomics at near-cellular resolution with slide-seqv2. Nat Biotechnol.

[17] Svensson V., Vento-Tormo R., Teichmann S.A. (2018). Exponential scaling of single-cell rna-seq in the past decade. Nat Protoc.

[18] Taniguchi Y., Choi P.J., Li G.-W. (2010). Quantifying e. coli proteome and transcriptome with single-molecule sensitivity in single cells. science.

[19] Vickovic S., Eraslan G., Salmén F. (2019). High-definition spatial transcriptomics for in situ tissue profiling. Nat Methods.

[20] Williams C.G., Lee H.J., Asatsuma T., Vento-Tormo R., Haque A. (2022). An introduction to spatial transcriptomics for biomedical research. Genome Med.

[21] Yan M., Sun Z., Wang J. (2023). Single-cell rna sequencing reveals distinct chondrocyte states in femoral cartilage under weight-bearing load in rheumatoid arthritis. Front Immunol.

[22] Zhang F., Wei K., Slowikowski K. (2019). Defining inflammatory cell states in rheumatoid arthritis joint synovial tissues by integrating single-cell transcriptomics and mass cytometry. Nat Immunol.

